# Office Notes

**Published:** 1871-02

**Authors:** W. H. Shadoan


### OFFICE NOTES.

BY W. H. SHADOAN, D. D. S.

[Continued from Page 537. Vol. XXIV.]

First right superior molar anterior approximal surface decayed. The decay in this case had extended so as to remove all the dentine beneath the anterior buccal cusp, up to within about two lines of the gum, so that when the cavity was prepared, the outer margin of the cavity extended slightly over the anterior buccal slope of the tooth.

In preparing the cavity the nerve was laid bare, which bled slightly. This was stopped by an application of a solution of carbolic acid and tannin. The lingual wall was nearly perpendicular, and at almost a right angle with the floor of the cavity. In this, as in the preceding case, the floor of the cavity was left by the decay slightly deeper within than at the cervical wall, which favored the preparation of the cavity. The retaining pits, three in number, were made one at each angle of the cervical wall, and the other up under the lingual cusp, which was then cut into a V shape, so as to afford one additional support to the filling. The edges of the cavity were then nicely dressed off with a file of suitable size and shape, when the rubber dam was adjusted and we were ready to proceed with the filling.

The point of exposure of the nerve was about a line from the buccal pit or retaining point. The cavity was as thoroughly dried with cotton, bibulous paper and punk as these agents would make it. The lingual pit was then filled a little more than full. The buccal pit was then treated in the same way and the two connected as in the first case. This brings the gold almost to the orifice of exposure of the pulp. I had already prepared a cap for the exposure, by taking a slip of three or four thicknesses of No. 4 gold foil and enveloped a small piece of asbestos, folding the gold over the asbestos so as to completely enclose it, making a pad about one-eighth of an inch square. This piece was then annealed, this does not injure the asbestos, and placed over the point of exposure, the edge coming in contact with the previously introduced gold, is then gently condensed with a foot instrument, No. 8, and the remainder of the cap gently pressed down on the dentine. I then continued the filling at the cervical wall as in the preceding case, being careful to use very light pressure over the point of exposure, until the filling was sufficiently advanced to give strength to the cap. Then with Nos. 8 and 9 the body of the filling was conducted, using Nos. 2 and 3 for the part next the walls. As soon as the filling has advanced from the cervical wall sufficiently to allow work on (what we will, for the want of a better name, call) the edge of the filling, we commence the use on that part with No. 10. It is readily seen that by the use of the Nos. 8, 9, 10, and 5, the filling can be made as perfect in every respect at the cervical wall as at any other part. If I use heavy gold, I use Nos. 14 and 15 for building, finishing with No. 10.

It is seen that the anterior buccal cusp is entirely gone; this is restored by the building up the filling to correspond to the original shape of the tooth.

[To be continued.]

---

Eds. Register:—The particulars of this interesting case were given me by Mr. Frost, aged thirty-six.

When about twenty-two years old, a soreness of the anterior inferior teeth was felt. This soreness continued with greater or less severity for two years, when the inflammation became extensive. The chin, neck, and thorax were involved and were much swollen. After a few days it broke opposite the first left inferior bicuspid, when the swelling subsided and nothing unusual was noticed for some time, when there was a repetition of the swelling, with the same result. When the swelling began the third time, the physician who examined it adroitly inserted the point of a lance in the *chin*. A discharge of pus followed, relieving at once his suffering and averting the threatened danger. For nearly a year the discharge continued, through a fistulous opening in the chin. No trouble was experienced when the opening was unobstructed, but when closed there was a tenderness of the left inferior central incisor. Being satisfied that this tooth was the cause of the trouble, he had it extracted, and the restoration to health of the parts, resulted without any subsequent treatment. C. H. R.

